# Transcriptome-wide identification of optimal reference genes for expression analysis of *Pyropia yezoensis* responses to abiotic stress

**DOI:** 10.1186/s12864-018-4643-8

**Published:** 2018-04-13

**Authors:** Dong Gao, Fanna Kong, Peipei Sun, Guiqi Bi, Yunxiang Mao

**Affiliations:** 1 Ministry of Education, Key Laboratory of Marine Genetics and Breeding (Ocean University of China), Qingdao, 266003 China; 2 Laboratory for Marine Biology and Biotechnology, Qingdao National Laboratory for Marine Science and Technology, Qingdao, 266237 China; 3 College of Marine Life Sciences, Ocean University of China, Qingdao, 266003 China

**Keywords:** Abiotic stress, *Pyropia yezoensis*, Reference genes

## Abstract

**Background:**

*Pyropia yezoensis*, a marine red alga, is an ideal research model for studying the mechanisms of abiotic stress tolerance in intertidal seaweed. Real-time quantitative polymerase chain reaction (RT-qPCR) is the most commonly used method to analyze gene expression levels. To accurately quantify gene expression, selection and validation of stable reference genes is required.

**Results:**

We used transcriptome profiling data from different abiotic stress treatments to identify six genes with relatively stable expression levels: *MAP, ATPase, CGS1, PPK, DPE2,* and *FHP*. These six genes and three conventional reference genes, *UBC*, *EF1-α*, and *eif4A*, were chosen as candidates for optimal reference gene selection. Five common statistical approaches (geNorm, ΔCt method, NormFinder, BestKeeper, and ReFinder) were used to identify the stability of each reference gene. Our results show that: *MAP*, *UBC*, and *FHP* are stably expressed in all analyzed conditions; *CGS1* and *UBC* are stably expressed under conditions of dehydration stress; and *MAP*, *UBC*, and *CGS1* are stably expressed under conditions of temperature stress.

**Conclusion:**

We have identified appropriate reference genes for RT-qPCR in *P. yezoensis* under different abiotic stress conditions which will facilitate studies of gene expression under these conditions.

**Electronic supplementary material:**

The online version of this article (10.1186/s12864-018-4643-8) contains supplementary material, which is available to authorized users.

## Background

*Pyropia yezoensis* (Ueda), previously known as *Prophyra yezoensis* [1], is a seaweed of economic importance. The gametophyte of this species has been widely cultivated and harvested in East Asia. *P. yezoensis* is an important seafood with annual production of over 1,100,000 t (in fresh weight) and an annual value of approximately US $1.5 billion (http://www.fao.org/fishery/statistics/en). *P. yezoensis* thrives in the upper intertidal zone. This is a harsh niche and during daily low tides, *P. yezoensis* is routinely exposed to high levels of light, dehydration, and extreme fluctuations in temperature and osmotic pressure due to the seawater to air transition. Blades can tolerate dehydration with water loss of up to 85%, but are metabolically active immediately upon rehydration [2]. These features make *P. yezoensis* an ideal model for studying the molecular mechanisms of intertidal seaweed stress-tolerance.

Precise quantification of expression fluctuations in genes involved in abiotic stress will further our understanding of stress-tolerance mechanisms in specific algae. RT-qPCR is widely used to assess gene expression and allows rapid and reliable quantification of transcripts expressed in low levels [3, 4]. However, for accurate quantification of transcripts in different spatial-temporal conditions, the crucial first step involves the selection of optimal reference genes. Housekeeping genes including actin (*ACT*), ubiquitin-binding protein (*UBC*), glyceraldehyde-3-phosphate dehydrogenase (*GAPDH*), translation initiation factor 4A (*eif4A*), elongation factor 1-α (*EF1-α*), and α-tubulin (*TUA*) are thought to be appropriate reference genes for the normalization of gene expression [5–7]. However, recent investigations have shown that these housekeeping genes may not be suitable for normalization of gene expression in all development stages or environmental conditions [8–10]. Moreover, it is difficult to completely normalize gene expression data from all types of samples using any single gene [11, 12]. Therefore, it is recommended that multiple reference genes be used to improve the reliability and accuracy of RT-qPCR results.

With the development of high-throughput sequencing technologies, RNA-sequencing (RNA-seq) provides a means to profile spatio-temporal transcriptomes [13, 14]. The rapid accumulation of transcriptome datasets presents a new strategy for the identification of novel sets of reference genes. For example, hundreds of candidates were mined from *Lycoris aurea* transcriptome datasets, and 14 were selected for further qPCR analysis after various abiotic stresses and from different tissues [15]. A similar approach was used to identify candidate reference genes from *Oxytropis ochrocephala* Bunge transcriptome datasets [16]. Twelve candidate genes were identified, and qPCR was used to analyze their expression levels following exposure to a range of abiotic stress conditions.

In *P. yezoensis*, the expression of seven housekeeping genes was quantified at different life-history stages and *GAPDH* was recommended as a potential internal control for gene expression studies [17]. In 2015, Kong validated the expression stability of six traditional housekeeping genes and recommended *ACT3*, *eIF4A*, and *EF1-α* as optimal *P. yezoensis* reference genes under conditions of stress [18]. Recently, accumulated *P. yezoensis* transcriptome data [19–21] has enabled us to identify sets of optimal reference genes. In this study, nine candidate reference genes were chosen for further analysis and optimal reference gene selection. These genes include three conventional reference genes (*UBC*, *EF1*-*α*, and *eIF4A*) and six genes (*MAP*, *ATPase*, *CGS1*, *PPK*, *DPE2*, and *FHP*) with relatively stable expression levels in transcriptome profiling data obtained under different abiotic stress conditions. Further, to validate the effectiveness of the selected reference genes, the expression levels of the Δ9 fatty acid desaturase (*PyOLE-1)* and oxygen-evolving enhancer protein 1 (*PyOEE-1)* target genes were quantified and compared with transcriptome profiling data.

These two genes, *PyOLE-1* and *PyOEE-1*, represent stress-responsive genes in algae. Under chilling and freezing temperature stress, *PyOLE-1*, which encodes a fatty acid desaturase, is up-regulated, to increase membrane fluidity [19]. *PyOEE-1* is a component of the oxygen evolving complex of photosystem II (PSII) and is, which were slowly down-regulated under drought stress in red algae [22].

Additionally, the best and worst reference genes (selected from *P. yezoensis* RZ58) were used to normalize the expression levels of these two target genes in two other genotypes of *P. yezoensis,* including a genetically pure line *(P. yezoensis* S21) and a cultural line (PyC-1), in order to demonstrate the applicability of our results within this species.

## Methods

### Plant materials and treatments

*Pyropia yezoensis* RZ58 is a genetically pure line established by clonal cultivation of an isolated single somatic cell and self-fertilization in the laboratory. Fresh leafy of RZ58 gametophytes were cultured in bubbling natural seawater with Provasoli’s enrichment solution medium under 50 μmol photons m^− 2^ s^− 1^ at 8 ± 1 °C and a 12:12 light:dark (L:D) photoperiod. Then the healthy gametophytes with sizes from 8 to 15 cm were treated with dehydration, rehydration, and cold and heat stress respectively. The same treatments were performed in S21 and PyC-1 too.

Gametophytes were subjected to dehydration and rehydration by exposing them to the air and then transferring them back to seawater. Algal samples under normal conditions were harvested before the dehydration treatment. Algal samples were also collected when the algae reached water loss levels of 20 ± 5%, 50 ± 5% and 70 ± 5% respectively. For rehydration, severely dehydrated algae were transferred back to normal conditions, and samples were collected after 0.5 h. Water loss was determined according to the method of Kim et al. [23]. All treatments were performed at 8 ± 1 °C and 50 μmol photons m^− 2^ s^− 1^.

For subjecting gametophytes to various temperature conditions, four temperature treatments were used: normal temperature (8 ± 1 °C), high temperature (24 ± 1 °C), chilling stress (0 ± 1 °C) and freezing temperature (− 8 ± 1 °C). Temperature stress was detected according to Sun et al. [19]. Three biological replicates were collected for each treatment and control, frozen in liquid nitrogen, and stored at − 80 °C prior to RNA isolation.

### RNA isolation and cDNA synthesis

Total RNA was extracted from samples using the Plant RNA Kit (Omega, USA). To eliminate DNA contamination, total RNA was digested with DNase I (Omega, USA) and purified according to the manufacturer’s protocol. RNA integrity was evaluated by 1% (*w*/*v*) agarose gel electrophoresis, and RNA concentration and purity was determined with a NanoDrop 2000 Spectrophotometer (NanoDrop Technologies, Thermo Scientific, USA). RNA samples with concentrations above 150 ng/μl and an A_260_/A_280_ ratio of 1.8–2.0 were used for cDNA synthesis.

An RNA aliquot of 1 μg was used for cDNA synthesis with PrimerScript™ RT regent Kit (TaKaRa Bio Inc., Dalian, China) according to the manufacturer’s protocol. The cDNA was diluted 10-fold with nuclease-free water for RT-qPCR.

### Selection of candidate reference genes and primer design

Due to a lack of *P. yezoensis* genome information, we generated a transcriptome for this species. Transcriptome sequencing of *P. yezoensis* (RZ58) was performed using Illumina paired-end sequencing technology on an Illumina Hi-Seq™ 2000 platform under the five treatments (control, dehydration, rehydration, cold, and heat). After assembly and annotation, expression profile data for each treatment was mapped to the transcriptome. The read counts of unigenes from different stress treatments were converted into fragments per kilobase of exon model per million mapped reads (FPKM values) using the RNA-Seq by Expectation Maximization software package [24].

The expression stability of each of the analyzed genes was calculated using the Pattern Gene Finder (PaGeFinder). PaGeFinder is a web-based server for the on-line detection of gene expression patterns from serial transcriptomic data generated by high-throughput technologies like microarray or next-generation sequencing. The dispersion measure (DPM) was introduced and implemented in PaGeFinder to evaluate the variability and degree of diversity of gene expression profiles. Most stable genes exhibit lower DPM values [25].

The transcriptome was screened for genes with credible protein annotation (Nr databases), appropriate expression levels (FPKM> 10), and a low dispersion measure (DPM ≤ 0.3) [25]; genes that met these criteria were deemed candidate reference genes (Table 1). Additionally, three commonly used reference genes, *UBC*, *EF1-α*, and *eIF4A*, were selected from the *P. yezoensis* transcriptome based on a previous study by Kong [18].

**Table 1:** Description of the candidate reference genes

Gene symbol	Gene ID	Gene length(bp)	NR description	Accession ID	MV(FPKM)	CV	DPM(dispersion measure)
MAP	TRINITY_DN10053_c0_g1_i1	1695	methionyl aminopeptidase [*Chondrus crispus*]	XP_005715371.1	104.792	0.130	0.129
ATPase	TRINITY_DN1508_c0_g1_i1	2582	AAA-type ATPase [*Galdieria sulphuraria*]	XP_005704492.1	36.383	0.136	0.134
CGS1	TRINITY_DN3362_c1_g1_i1	2083	Cystathionine gamma-synthase, Rhodoplastic CGS1 [*Chondrus crispus*]	XP_005718068.1	175.309	0.166	0.164
PPK	TRINITY_DN8109_c0_g1_i1	3034	polyphosphate kinase, partial [*Pyropia yezoensis*]	CAM33394.1	24.197	0.166	0.164
DPE2	TRINITY_DN10209_c0_g1_i1	2847	Disproportionating Enzyme type 2 [*Chondrus crispus*]	XP_005714071.1	76.424	0.167	0.165
FHP	TRINITY_DN7986_c2_g1_i1	1797	fumarate hydratase precursor [*Chondrus crispus*]	XP_005718019.1	83.279	0.170	0.168
UBC	TRINITY_DN9056_c0_g3_i1	530	putative ubiquitin-conjugating enzyme [*Pyropia yezoensis*]	ACI47322.1	374.296	0.649	> 0.3
EF 1-α	TRINITY_DN9169_c0_g1_i2	1632	RecName: Full = Elongation factor 1-alpha; Short = EF-1-alpha	Q8LPC4.1	1079.455	0.475	> 0.3
eif4A	TRINITY_DN16387_c0_g1_i1	1285	putative eukaryotic translation initiation factor 4A [*Pyropia yezoensis*]	ACJ22452.1	1149.390	0.779	> 0.3

Specific primers were designed using Primer5 software based on the sequences of these unigenes (Table 2). The criteria for primer design were as follows: primer lengths of 17–24 bp, GC content of 50–66%, melting temperature of 58–61 °C, and amplicon lengths of 100–200 bp.

**Table 2:** Genes and primer sets for RT-qPCR

Gene name	Gene ID	Gene symbol	Prime sequence (forward/reverse)	Size(bp)	RT-qPCR Efficiency^a^	Error^b^
Methionyl aminopeptidase	TRINITY_DN10053_c0_g1_i1	MAP	TGGGTAGGAAGTGGGGCT	200	1.946	0.0793
			CGTGGTAGGTCGGTAGGC			
AAA-type ATPase	TRINITY_DN1508_c0_g1_i1	ATPase	CGACGAGATTGACGCA	132	2.014	0.0744
			GTCGCCCCAATCACAA			
Cystathionine gamma-synthase, Rhodoplastic	TRINITY_DN3362_c1_g1_i1	CGS1	CTACGGACACCAAGAAACG	106	1.947	0.121
			CTCGGTTGGCTGGGTAA			
polyphosphate kinase, partial	TRINITY_DN8109_c0_g1_i1	PPK	GTGTCTGGTCCACGCTC	160	1.98	0.101
			CACGAGGTGCTGACTGAG			
Disproportionating Enzyme type 2	TRINITY_DN10209_c0_g1_i1	DPE2	CACGGAAGGTAGGAAAGGA	204	1.992	0.0582
			AGGTGGGTGTTGGGGTT			
fumarate hydratase precursor	TRINITY_DN7986_c2_g1_i1	FHP	TAATGTGCGAAAAGGCGG	146	1.973	0.117
			CGTGAACAAGTCCCAGTCCT			
ubiquitin-conjugating enzyme	TRINITY_DN9056_c0_g3_i1	UBC	CGCTGACCGTTTCCAAG	112	1.99	0.0598
			CGACTGCGGTTGGACTT			
elongation factor	TRINITY_DN9169_c0_g1_i2	EF	TGCGAGTCAACCAGGAG	175	1.96	0.153
			GCCTCAAGAAACACCCTA			
translation initiation factor 4 A	TRINITY_DN16387_c0_g1_i1	eif4A	ATGGACCAGAAGGACCG	139	1.951	0.144
			TCGTGGGCAGGTCATAG			

^a^ The RT-qPCR amplification efficiency for each primer were determined by LightCycle®480 gene scanning software (version 1.5)

^b^ The Error value of amplification efficiency for each primer were determined by LightCycle®480 gene scanning software (version 1.5) and an acceptable vaule should be< 0.2

### RT-qPCR analysis

RT-qPCR was conducted in 96-well plates in a LightCycler 480 (Roche Molecular Biochemicals, Mannheim, Germany). The reaction mix contained 2 μl diluted cDNA, 10 μl LightCycler®480 SYBR Green I Master (Roche, Germany), 0.6 μl of each primer, and ddH_2_O in a final volume of 20 μL. Three biological replicates were performed for each treatment. Three technical replicates of each biological replicate as well as a no-template control were also performed. RT-qPCR cycling parameters were as follows: 95 °C for 5 min, followed by 45 cycles of 95 °C for 10 s, 58 °C for 10 s, and 72 °C for 20 s. To confirm the specificity of each primer, a melting-curve analysis was included from 65 °C to 95 °C. The mean amplification efficiency of each primer pair was checked by the LightCycle®480 gene scanning software (version 1.5).

### Data analysis

The three most commonly used software tools, geNorm, NormFinder, and BestKeeper, were used in conjunction with the comparative ΔCt method to calculate and identify the expression level stability of each candidate reference gene.

The geNorm algorithm [26] calculates the expression stability value (M-value) and pairwise variation (Vn/n + 1) for all candidate genes. Lower M-values reflect a greater level of gene expression stability. The Vn/n + 1 value determines the optimal number of reference genes for accurate normalization. A cut-off value of Vn/n + 1 < 0.15 indicates that an additional reference genes make no significant contribution to the normalization.

The NormFinder program [27] is based on an ANOVA model, and calculates a stability value (SV) for evaluating expression variation when using reference genes for normalization with a lower SV indicating higher stability.

BestKeeper [28] is an Excel-based tool that uses pair-wise correlations. BestKeeper calculates three variables for the expression level of all candidate genes: the standard deviation, coefficient of correlation (r), and coefficient of variance (CV). The BestKeeper index is established based on the combination of the mean of Ct values for each sample across all candidate genes. Subsequently, each candidate gene is tested in a pair-wise manner via Pearson correlation coefficients, the coefficient of determination (r^2^), and the *P*-value. The most stable gene exhibits the lowest CV ± SD (standard deviation) value, and genes with an SD value greater than one are deemed unacceptable and should be excluded.

The comparative ΔCt method depends on pairwise comparisons [29], which calculate the mean and SD of each pair candidate genes and the average SD of each gene. The gene with lowest average SD is considered the most stable reference gene.

### Comprehensively ranking the candidate genes

A comprehensive stability value for each gene was produced using ReFinder (http://150.216.56.64/referencegene.php) [30] based on the four computational programs, NormFinder, BestKeeper, GeNorm, and comparative ΔCt. The Ct value of each gene was input directly and the geometric mean of each gene was calculated to arrive at its overall final ranking. A lower geometric mean of ranking value indicates more stable expression.

### Experimental validation of the reference genes

The expression patterns of the two target genes *PyOLE-1* and *PyOEE-1* were analyzed using the most and least stable reference gene sets after normalization across two experimental sets temperature stress (for *PyOLE-1*) and drought stress (for *PyOEE-1*). To validate the results, the expression levels of the target genes based on RT-qPCR were compared with the FPKM values derived from the RNA-seq data for each sample. Moreover, the relative expression levels of each target gene were compared using a single reference gene as well as the most stable reference genes to determine whether the inclusion of multiple reference genes improves the reliability and accuracy of RT-qPCR results.

Finally, we also quantified the expression patterns of these target genes in two another genotypes of *P. yezoensis* (S21 and PyC-1) using the most and least stable reference gene sets.

## Results

### Global transcriptome assembly and function annotation

A total of 1.72 × 10^7^ quality paired-end reads were obtained after filtering out low-quality data (tags containing adaptors). The GC content of the transcriptome was 67.74%. After assembly and annotation, a total of 19,643 unigenes with a mean length of 779.5 bp and an N50 value of 1149 bp were obtained. To assign accurate annotation information to all unigenes, the NCBI non-redundant protein (Nr) database was interrogated, and a total of 13,160 unigenes (67%) were annotated.

### Selection of candidate reference genes in *P. yezoensis* and specificity and efficiency of PCR amplification

As shown in Additional file 1: Figure S1, the expression stabilities of all transcripts were evaluated by PaGeFinder the results showed that only 2060 unigenes (326 unigenes marked by asterisk whose DPM values were lower than 0.2 and all of novel reference genes derived from them; the DPM value of 1734 unigenes were located in the range from 0.2 to 0.3) can be used to further reference genes selection (DPM < 0.3). Then, we removed some transcripts which did not have a credible function annotation or whose expression level is too low (FPKM< 10) [15, 16]. Finally, 865 unigenes were retained.

*MAP*, *ATPase*, *CGS1*, *PPK*, *DPE2*, and *FHP* were selected from these unigenes based on the ranked order of the DPM values from smallest to largest. Three commonly used reference genes, *UBC*, *EF1-α,* and *eif4A*, were selected from the transcriptome directly [19].

Primers were designed for each of the nine genes and their specificities were confirmed by agarose gel electrophoresis, and melting curves analysis, which showed single amplicon of the expected size and single peak melting curve (Additional file 2: Figure S2). Meanwhile, we also sequenced all PCR products to ensure that only the intended target was being amplified (Table 2). RT-qPCR products ranged from 137 to 213 bp and the mean PCR efficiency for each gene ranged from 1.946 to 2.014.

### Cq values of candidate reference genes

RNA transcript levels of the nine reference genes were assessed in conditions of dehydration and temperature stress. The raw Cq values for the reference genes are shown in Additional file 3: Figure S3.

According to the summary showed in Additional file 3: Figure S3A, the raw Cq values of dehydration samples were between 18.90 and 33.28 for *UBC* and *eif4A*, respectively. Mean Cq values ranged from 20.04 to 26.79 for *UBC* and *PPK*, respectively. *MAP*, *PPK*, and *DPE2* had low expression levels with high Cq values, and *CGS1*, *FHP*, and *eif4A* showed moderate expression levels. *UBC*, *EF1-α*, and *ATPase* demonstrated high expression levels with low Cq values (20.04, 20.88, and 23.13 respectively). The SDs of the Cq values for *UBC* (20.04 ± 0.27) and *CGS1* (24.82 ± 0.16) were much lower than those of *DPE2* (25.26 ± 0.97) and *eif4A* (23.95 ± 0.99). Under temperature stress, the raw Ct values ranged from 17.80 to 31.53 for *UBC* and *DPE2*, respectively (Additional file 3: Figure S3B). Of the nine candidate reference genes, *CGS1*, *PPK*, and *DPE2* were observed to have the lowest expression levels with Ct values of 25.84, 25.36, and 28.56, respectively. Ct values for *ATPase*, *UBC*, and *EF1-α* were 21.54, 20.00, and 21.12, respectively, indicating that transcripts of these genes were abundant in samples under temperature stress. The least variable reference genes were *MAP* and *ATPase* with SD values of 0.29 and 0.47, respectively. Conversely, *DPE2* and *eif4A* were the most variable genes with SD values of 1.30 and 1.53, respectively.

### Expression stability of candidate reference genes

To identify optimal reference genes for the experimental conditions used, four statistical approaches were employed. The M-values of nine reference genes were calculated and the stability of each candidate reference gene was ranked by the M-value calculated using geNorm. Genes with the lowest M-values are considered to have the most stable expression with an M-value less than 0.5 denoting stable gene expression [31]. geNorm analysis showed that *CGS1* and *FHP* shared the lowest M-value of 0.219, and were regarded as the best reference genes for dehydration stress (Additional file 4: Figure S4A). Under conditions of temperature stress, *MAP* and *ATPase* were the reference genes with the greatest expression stability (Additional file 4: Figure S4B). When considering all treatments, the M-based ranking of the reference genes examined, from most (lowest M value) to least stable (highest M value), was: *MAP*, *UBC*, *FHP*, *EF1-α*, *CGS1*, *ATPase*, *PPK*, *eifA*, and *DPE2* (Table 3 and Additional file 4: Figure S4C).

**Table 3:** geNorm ranking for the 9 candidate reference genes

Rank	All stress		Dehydration stress		Temperature stress	
	Gene	M value	Gene	M value	Gene	M value
1	MAP	0.478	CGS1	0.219	MAP	0.485
2	UBC	0.478	FHP	0.219	ATPase	0.485
3	FHP	0.592	PPK	0.314	EF1-α	0.544
4	EF1-α	0.646	UBC	0.357	UBC	0.583
5	CGS1	0.744	MAP	0.466	CGS1	0.628
6	PPK	0.904	EF1-α	0.566	FHP	0.65
7	ATPase	1	eif4A	0.67	PPK	0.868
8	eif4A	1.111	DPE2	0.79	DPE2	1.045
9	DPE2	1.376	ATPase	0.895	eif4A	1.188

M value: expression stability value

In the dehydration stress subset, the V2/3 value was 0.115, suggesting that two reference genes should be used for normalization. In the temperature stress subset, the V3/4 value was lower than 0.15 indicating that only three reference genes were necessary. Additionally, when all samples were considered, the pairwise variation V3/4 value was the lowest (0.153) but still above 0.15 (Additional file 5: Figure S5 and Additional file 6: Table S1).

Based on normalization factor calculation, NormFinder ranked the candidate reference genes according to their minimal combined inter- and intra-treatment expression variation. According to the stability value calculated with the NormFinder algorithm, *UBC*, *CGS1*, and *FHP* were the most reliable references genes for dehydration treatments, and *UBC*, *MAP*, and *CGS1* were the optimal reference genes for conditions of heat and cold stress. When both dehydration and temperature treatments were considered together, the three most reliable reference genes were *UBC*, *MAP* and *EF1-α* with stability values of 0.254, 0.373 and 0.406 respectively (Table 4).

**Table 4:** Expression stability of the 9 candidate reference genes calculated by NormFinder

Rank	All stress		Dehydration stress		Temperature stress	
	Gene	Stability	Gene	Stability	Gene	Stability
1	UBC	0.254	UBC	0.209	UBC	0.173
2	MAP	0.373	CGS1	0.257	MAP	0.293
3	EF1-α	0.406	FHP	0.315	CGS1	0.402
4	FHP	0.408	MAP	0.342	EF1-α	0.424
5	CGS1	0.491	EF1-α	0.365	ATPase	0.476
6	PPK	0.708	PPK	0.370	FHP	0.505
7	eif4A	0.761	eif4A	0.527	DPE2	0.718
8	ATPase	0.783	DPE2	0.589	PPK	0.847
9	DPE2	1.246	ATPase	0.646	eif4A	0.972

BestKeeper analysis determined stable reference gene candidates based on the Ct values of each gene, SD, and CV. Genes with a SD greater than one are considered unstable. Under conditions of dehydration stress *CGS1* (0.63 ± 0.16) and *FHP* (0.67 ± 0.16) were the most stable genes. While under conditions of temperature stress, *MAP*, *ATPase*, and *UBC* were the most suitable reference genes. Combining all abiotic stress treatment conditions revealed that *UBC*, *MAP*, and *FHP* had CV ± SD values of 1.68 ± 0.43, 2.08 ± 0.41, and 2.04 ± 0.49 respectively, and were regarded as the most appropriate reference genes for normalization (Table 5).

**Table 5:** Expression stability of 9 candidate reference genes calculated by BestKeeper

Rank	All stress			Dehydration stress			Temperature stress		
	Gene	SD	CV	Gene	SD	CV	Gene	SD	CV
1	MAP	0.43	1.68	CGS1	0.16	0.63	MAP	0.29	1.16
2	UBC	0.41	2.08	FHP	0.16	0.67	ATPase	0.47	2.19
3	FHP	0.49	2.04	PPK	0.27	1	UBC	0.52	2.68
4	PPK	0.67	2.55	UBC	0.27	1.39	CGS1	0.74	2.86
5	CGS1	0.69	2.74	MAP	0.5	1.94	PPK	0.75	2.93
6	EF1-α	0.7	3.34	EF1-α	0.68	3.28	EF1-α	0.69	3.28
7	ATPase	0.81	3.6	DPE2	0.97	3.86	FHP	0.88	3.71
8	eif4A	1.17	5.07	ATPase	0.98	4.28	DPE2	1.3	4.57
9	DPE2	1.68	6.34	eif4A	0.99	4.27	eif4A	1.53	6.69

By comparing the differential expression of ‘gene pairs’, the ΔCt method identifies stably co-expressed gene pairs when the ΔCt value of two genes remains constant across different samples [32]. The boxplot of ΔCt values for each ‘gene pair’ is shown in Additional file 7: Figure S6. Table 6, the results showed that *UBC* (mean SD = 0.948), *MAP* (mean SD =1.009), and *FHP* (mean SD = 1.048) were the most reliable reference genes under all analyzed conditions; *UBC* and *CGS1* are stably expressed under conditions of dehydration stress; and *UBC*, *MAP*, and *ATPase* are stably expressed under conditions of temperature stress with mean SD values of 0.885, 0.886 and 0.920 respectively.

**Table 6:** Expression stability of 9 candidate reference genes calculated by ΔCt method

Rank	All stress		Dehydration stress		Temperature stress	
	Gene	MeanSD	Gene	MeanSD	Gene	MeanSD
1	UBC	0.948	UBC	0.573	UBC	0.885
2	MAP	1.009	CGS1	0.596	MAP	0.886
3	FHP	1.048	FHP	0.666	ATPase	0.920
4	EF1-α	1.079	MAP	0.680	EF1-α	0.935
5	CGS1	1.190	PPK	0.739	CGS1	0.945
6	ATPase	1.347	EF1-α	0.799	FHP	1.019
7	eif4A	1.435	eif4A	0.852	DPE2	1.636
8	PPK	1.495	ATPase	1.050	PPK	1.681
9	DPE2	2.338	DPE2	1.073	eif4A	1.702

### Comprehensive stability analysis of reference genes

ReFinder integrates the four statistical approaches used to compare and rank the candidate reference genes. In all abiotic stress treatments, ReFinder ranked the candidate reference genes from the highest to the lowest stability as: *UBC* > *MAP* > *FHP* > *EF1-α* > *CGS1* > *ATPase* > *eif4A* > *DPE2* (Table 7). Under conditions of temperature stress, *MAP*, *UBC*, and *CGS1* were the three most stable reference genes analyzed, while under dehydration stress the most stable reference genes were *CGS1*, followed by *UBC* and *FHP*. The overall ranking showed that *UBC* and *MAP* were the most reliable reference genes in all different abiotic stress conditions, while *DPE2* and *eif4A* were the least reliable.

**Table 7:** Comprehensive ranking of the expression stability of 9 candidate reference genes

Method	1	2	3	4	5	6	7	8	9
A.RANKING ORDER UNDER ALL STRESS(BETTER-GOOD-AVERAGE)									
geNorm	MAP/UBC		FHP	EF 1-α	CGS1	PPK	ATPase	eif4A	DPE2
NormFinder	UBC	MAP	EF 1-α	FHP	CGS1	PPK	eif4A	ATPase	DPE2
BestKeeper	MAP	UBC	FHP	PPK	CGS1	EF 1-α	ATPase	eif4A	DPE2
Δct method	UBC	MAP	FHP	EF 1-α	CGS1	ATPase	eif4A	PPK	DPE2
Comprehensive ranking	UBC	MAP	FHP	EF 1-α	CGS1	PPK	ATPase	eif4A	DPE2
B.RANKING ORDER UNDER DEHYDRATION STRESS (BETTER-GOOD-AVERAGE)									
geNorm	CGS1/FHP		PPK	UBC	MAP	EF 1-α	eif4A	DPE2	ATPase
NormFinder	UBC	CGS1	FHP	MAP	EF 1-α	PPK	eif4A	DPE2	ATPase
BestKeeper	CGS1	FHP	PPK	UBC	MAP	EF 1-α	DPE2	ATPase	eif4A
Δct method	UBC	CGS1	FHP	MAP	PPK	EF 1-α	eif4A	ATPase	DPE2
Comprehensive ranking	CGS1	UBC	FHP	PPK	MAP	EF 1-α	eif4A	DPE2	ATPase
C.RANKING ORDER UNDER TEMPERATURE STRESS (BETTER-GOOD-AVERAGE)									
geNorm	MAP/ATPase		EF 1-α	UBC	CGS1	FHP	PPK	DPE2	eif4A
NormFinder	UBC	MAP	CGS1	EF 1-α	ATPase	FHP	DPE2	PPK	eif4A
BestKeeper	MAP	ATPase	UBC	CGS1	PPK	EF 1-α	FHP	DPE2	eif4A
Δct method	UBC	MAP	ATPase	EF 1-α	CGS1	FHP	DPE2	PPK	eif4A
Comprehensive ranking	MAP	UBC	CGS1	ATPase	EF 1-α	FHP	PPK	DPE2	eif4A

### Reference genes validation

**Fig. 1:**
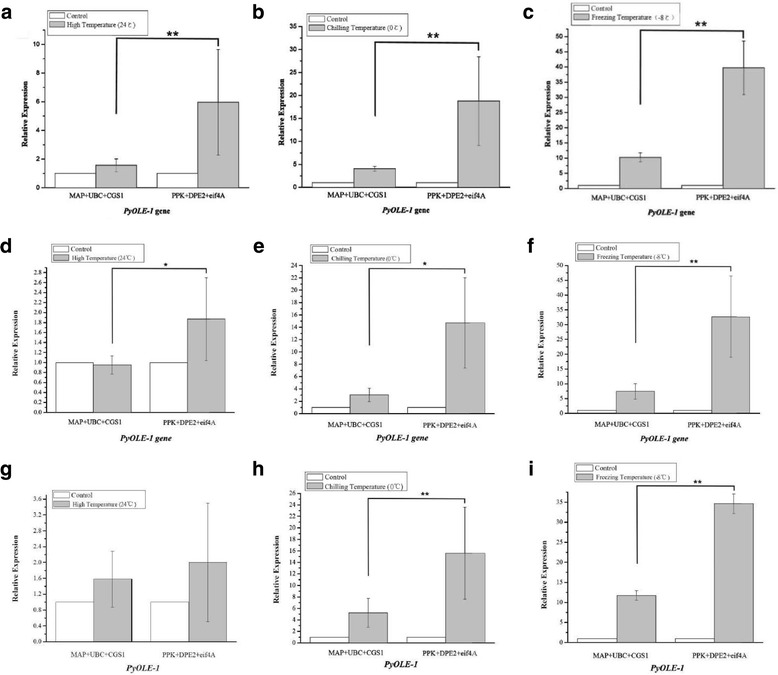
Normalized expression level of *PyOLE-1* gene in different genotypes in temperature stress. (**a**, **b**, **c**) Relative quantification of *PyOLE-1* gene expression using the best stable reference genes (*MAP, UBC* and *CGS1*) and the least stable genes (*PPK, DPE2* and *eif4A*) under temperature stress in RZ58. **d**, **e**, **f** Relative quantification of *PyOLE-1* gene expression using the best stable reference genes (*MAP, UBC* and *CGS1*) and the least stable genes (*PPK, DPE2* and *eif4A*) under temperature stress in S21. **g**, **h**, **i** Relative quantification of *PyOLE-1* gene expression using the best stable reference genes (*MAP, UBC* and *CGS1*) and the least stable genes (*PPK, DPE2* and *eif4A*) under temperature stress in PyC-1. The average Ct value was calculated from three biological and technical replicates and used for relative expression analyses. Error bars indicate standard errors. The statistical significance was showed by one or two asterisks (*P* value is lower than 0.05 or 0.01) respectively

**Fig. 2:**
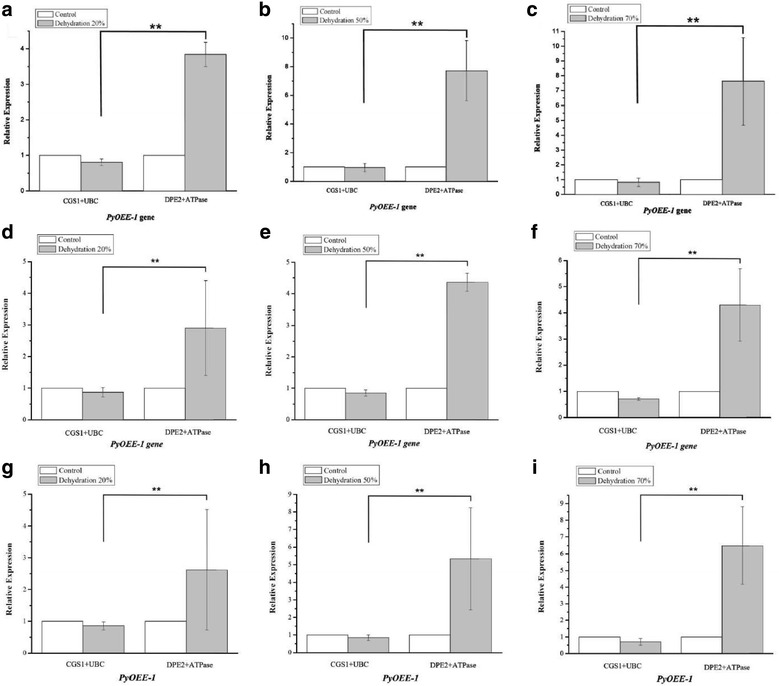
Normalized expression level of *PyOEE-1* gene in different genotypes in dehydration stress. (**a**, **b**, **c**) Relative quantification of *PyOEE-1* gene expression using the best stable reference genes (*CGS1* and *UBC*) and the least stable genes *(DPE2* and *ATPase*) under dehydration stress in RZ58. **d**, **e**, **f** Relative quantification of *PyOEE-1* gene expression using the best stable reference genes (*CGS1* and *UBC*) and the least stable genes (*DPE2* and *ATPase*) under dehydration stress in S21. **g**, **h**, **i** Relative quantification of *PyOEE-1* gene expression using the best stable reference genes (*CGS1* and *UBC*) and the least stable genes (*DPE2* and *ATPase*) under dehydration in PyC-1. The average Ct value was calculated from three biological and technical replicates and used for relative expression analyses. Error bars indicate standard errors. The statistical significance was showed by one or two asterisks (P value is lower than 0.05 or 0.01) respectively

**Fig. 3:**
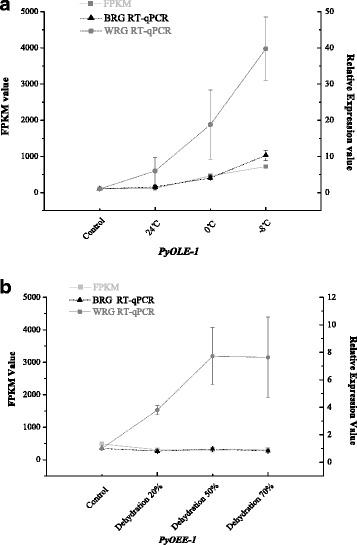
Comparison of normalized expression level of target genes in RT-qPCR and FPKM value under temperature and dehydration stress respextively. (**a**) For temperature conditions, FPKM values of *PyOLE-1* were showed in this figure and evaluated it by the Y-axis of left, and the right Y-axis were used to indicate the relative quantifications of *PyOLE-1*, normalized by the most stable reference genes *(MAP, UBC* and *CGS1*) and the worst stable reference genes (*PPK, DPE2* and *eif4A*). **b** For dehydration conditions, FPKM values of *PyOEE-1* were showed in this figure and evaluated it by the Y-axis of left, and the right Y-axis were used to indicate the relative quantifications of *PyOEE-1*, normalized by the most stable reference genes *(CGS1* and *UBC*) and the worst stable reference genes (*DPE2* and *ATPase*)

**Fig. 4:**
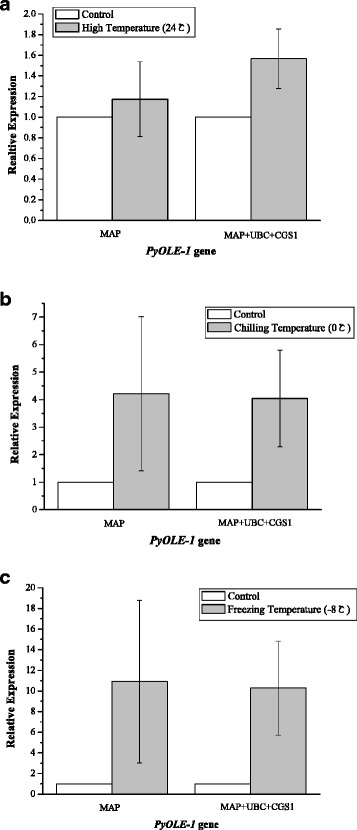
Comparison of Normalized expression level of *PyOLE-1* gene between single and the best reference genes under temperature stress. (**a**) Relative quantification of *PyOLE-1* gene expression using the single reference gene *MAP* and the best stable reference genes (*MAP, UBC* and *CGS1*) under high temperature (**a**), chilling temperature (**b**) and freezing temperature (**c**) respectively. The average Ct value was calculated from three biological and technical replicates and used for relative expression analyses

**Fig. 5:**
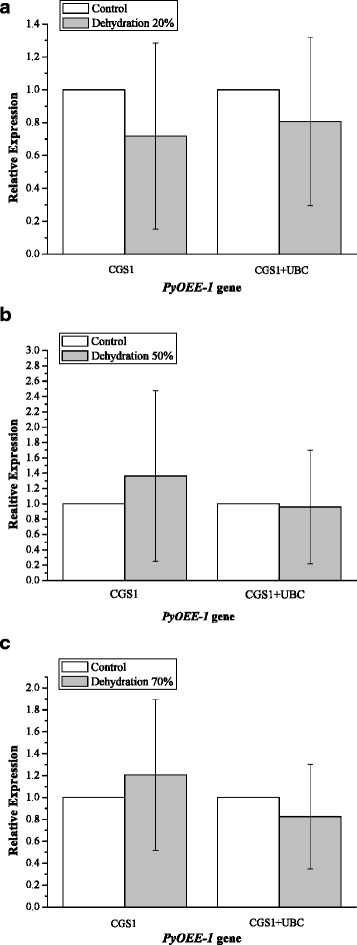
Comparison of Normalized expression level of *PyOEE-1* between single and the best reference genes under dehydration stress. (**a**) Relative quantification of *PyOEE-1* gene expression using the single reference gene *CGS1* and the best stable reference genes (*CGS1* and *UBC*) under water loss rate 20% (A), 50% (**b**) and 70%(**c**) respectively. The average Ct value was calculated from three biological and technical replicates and used for relative expression analyses

Under conditions of temperature stress, at 24 °C, 0 °C, and − 8 °C, *PyOLE-1* expression was up-regulated 1.50-fold, 4.05-fold, and 10.28-fold respectively, when using the most stable reference genes (*MAP*, *UBC*, and *CGS1*). Using the least stable reference genes, *PPK*, *DPE2*, and *eif4A*, resulted in overestimation of *PyOLE-1* expression was overestimated at 5.97-fold, 18.78-fold, and 39.76-fold for conditions of 24 °C, 0 °C, and − 8 °C, respectively (Fig. 1a, b, c). Similarly, under conditions of dehydration, with water loss rates of 20%, 50%, and 70%, *PyOEE-1* expression was down-regulated 0.81-fold, 0.96-fold, and 0.82-fold, respectively, when normalized using the two stable genes (*CGS1* and *UBC*). In contrast, the expression levels of *PyOEE-1* were up-regulated 3.80-fold, 7.72-fold, and 7.63-fold respectively, when the least stable reference genes (*ATPase* and *DPE2*) were used (Fig. 2a, b, c). Next, we compared these RT-qPCR results with those derived from the RNA-seq-based expression profiling. As shown in Fig. 3, the relative expression levels of *PyOLE-1* quantified by the best reference genes were more consistent with the RNA-seq-based expression patterns of *PyOLE-1* under temperature stress (up-regulated 1.03 fold, 4.19 fold and 6.50 fold). Under drought conditions, the RT-qPCR results, when normalized by the best reference genes, were also more similar to the RNA-seq-based results (down-regulated 0.63 fold, 0.60 fold and 0.67 fold). Furthermore, we compared the difference between the relative expression levels when using a single reference gene and those obtained using the best reference genes. Under conditions of temperature stress, similar *PyOLE-1* expression levels were observed when either a single reference gene or multiple reference genes were used (Fig. 4). At 24 °C, 0 °C and − 8 °C, when only *MAP* was used as a reference gene, the relative expression of *PyOLE-1* was up-regulated by 1.17-fold, 4.21-fold and 10.92-fold respectively. Similarly, *PyOLE-1* was up-regulated by 1.56-fold, 4.05-fold and 10.28-fold respectively, when several stable reference genes (*MAP, UBC* and *CGS1*) were employed to calculate the relative expression of *PyOLE-1*. Under conditions of 20%, 50% and 70% dehydration, however, the *PyOEE-1* expression levels were down regulated by 0.72-fold, up-regulated 1.36-fold, and 1.21-fold when using a single reference gene (*CGS1*), while relative expression levels of *PyOEE-1* were down-regulated: 0.81-fold, 0.96-fold and 0.82-fold respectively, when *CGS1* and *UBC* were both used as reference genes (Fig. 5). Additionally, to confirm whether our reference genes could be applied to other experimental models of *P. yezoensis* under the same abiotic stress conditions, two other genotypes of this species (S21 and PyC-1) were treated with the same temperature and drought stress like conditions as RZ58. Then, we used the most and lest stable reference genes (*MAP*, *UBC, CGS1, PPK, DPE2* and *eif4A* for temperature conditions and *CGS1, UBC, DPE2* and *ATPase* for dehydration conditions) to normalize the relative expression levels of *PyOLE-1* and *PyOEE-1*.

For temperature stress, the similar with RZ58, relative expression levels of *PyOLE-1* were observed in S21 and PyC-1 (Fig. 1d, e, f and g, h, i), when the best reference genes were used for quantification. In S21, *PyOLE-1* was up regulated: 1.58-fold, 5.25-fold and 11.74-fold at 24 °C, 0 °C, and − 8 °C, respectively, while, in PyC-1, *PyOLE-1* was up-regualted 0.95-fold, 3.03-fold and 7.45-fold, respectively. In contrast, the expression level of *PyOLE-1* was overestimated by 2.03-fold, 15.58-fold and 34.62-fold respectively, in PyC-1 when the least stable reference genes were used.

Under conditions of drought, with water loss rates of 20%, 50% and 70%, *PyOEE-1* was down-regulated 0.87-fold, 0.85-fold and 0.71-fold in S21 and 0.87-fold, 0.85-fold and 0.70-fold in PyC-1, respectively (Fig. 2d, e, f and g, h, i) when the best reference genes were used. Using the worst reference genes, however, the expression levels of *PyOEE-1* were up-regulated at 2.94-fold, 4.36-fold and 4.30-fold in S21 and 2.62-fold, 5.33-fold and 6.50-fold in PyC-1, respectively.

## Discussion

RT-qPCR is a highly sensitive technique used in a wide range of applications. Therefore, the selection and use of suitable reference genes is a prerequisite for the accurate quantification of gene expression levels. Based on current researches, transcriptomic profile data is a reliable source for exploration of suitable reference genes for specific experimental conditions [15, 16, 33]. In this study, six candidate reference genes were selected from transcriptome of *P. yezoensis* by some criteria including credible protein annotation (Nr databases), appropriate expression levels (FPKM> 10), and a low dispersion measure (DPM ≤ 0.3).

On the other hand, traditionally, housekeeping genes have been widely used as reference genes due to their ubiquitous expression in different spatial-temporal conditions. However, recent studies show that expression levels of some classic reference genes are not as stable as previously thought [8, 34]. Here, three housekeeping genes (*UBC*, *EF1-α*, and *eif4A*) were selected to evaluate their suitability as reference genes for qPCR following abiotic stress. *UBC*, an ubiquitin-conjugating enzyme gene, exhibited stable expression in each group examined, whilst the *EF1-α* was not one of the top three most stable genes in any of the experimental conditions. Nevertheless, *eif4A* expression was variable in all experimental subsets, and especially in the temperature subset. Therefore, we should take results with some caution when housekeeping genes have been directly used as reference genes for expression normalization in the absence of validation.

Gene expression can be highly tissue-specific and often varies based on the physiological status of the organism or experimental treatments. Therefore, the simultaneous use of several reference genes could decrease the probability of biased normalization [35, 36]. In addition to identifying the most stable reference genes, geNorm results suggested the optimum pair of genes with the least amount of variation in their expression ratios.

To validate selected reference genes, the relative expression levels of the target genes according to RT-qPCR were compared with those derived from RNA-seq-based gene expression profiling. As shown in Additional file 2: Figure S2, the RT-qPCR results quantified using best reference genes were more consistent with the RNA-seq-based target genes expression patterns.

Our results also indicated that multiple internal references are necessary for the accurate study of gene expression under various experimental conditions. Indeed, a single reference gene can be insufficient to accurately normalize expression data, or can lead to erroneous interpretation. The optimal number of reference genes required for RT-qPCR analysis has been ongoing discussion [26]. A threshold value of V < 0.15 was suggested for normalization. Our results demonstrated that two genes (*CGS1* and *UBC*) were suitable for normalization under dehydration stress, whilst three genes (*MAP*, *UBC*, and *CGS1*) were optimal for temperature stress. In addition, geNorm showed that the V3/4 value was the lowest among all treatment samples but that it was still above 0.15. According to several reports, the threshold V value (pairwise variation) of 0.15 should not be considered as an absolute cutoff but rather a suggested one. Some studies have even reported higher V values in some species, and the threshold used is thus dependent on a consideration of the research purpose [37, 38]. Considering our results and the practical feasibility, three genes (*UBC*, *MAP*, and *FHP*) were shown to be appropriate for gene expression normalization when all samples were analyzed in combination.

**Fig. 6:**
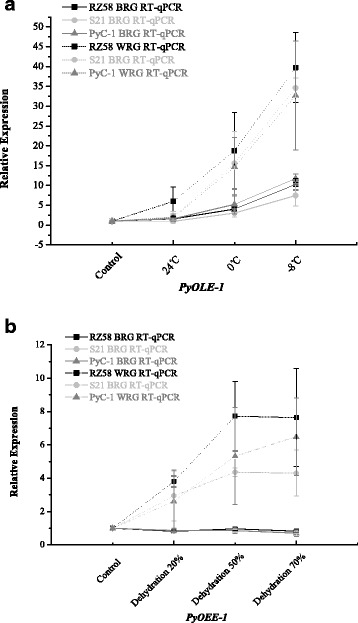
Comparison of relative expression level of target genes among different genotypes. (**a**) The relative expression level of *PyOLE-1* when using the best (BRG) and the worst reference genes (WRG) in different genotypes (RZ58, S21 and PyC-1). (**b**) The relative expression level of *PyOEE-1* when using the best (BRG) and the worst reference genes (WRG) in different genotypes (RZ58, S21 and PyC-1)

Additionally, in order to confirm that our results are more generally applicable across the species, other two genotypes of *P. yezoensis* were treated with the same abiotic stress conditions and the relative expression patterns of *PyOLE-1* and *PyOEE-1* were then quantified using the best reference and the worst reference genes. We obtained results similar to those obtained for line RZ58, and these results were also consistent with the characteristics of the target genes in algae under abiotic stress (Fig. 6).

## Conclusions

In this study, six candidate genes were selected form the transcriptome of *P. yezoensis* to search several appropriate reference genes for using in RT-qPCR. And this study also demonstrated that the use of housekeeping genes as reference genes for normalization of expression data should be validated. Based on these results, we identified optimal sets of reference genes to accurately normalize and quantify gene expression under abiotic stress conditions in *P. yezoensis*. We also compared their difference of expression levels between RNA-seq data and RT-qPCR data in different treatments. The result showed that the RNA-seq data is reliable to valuing the expression stability of genes. Further, our results also indicated that multiple reference genes are necessary for accurate study of gene expression in different treatments, such as *CGS1* and *UBC* were suitable for dehydration stress. And, the similarly expression patterns of *PyOLE-1* and *PyOOE-1* were observed in two other genotypes of *P. yezoensis* confirmed that our identified reference genes more generally applicable across the species. In summary, these reference genes will facilitate further research towards elucidating the molecular mechanisms of stress-tolerance in this economically important species*.*

## Additional files

Additional file 1: **Figure S1.** Stability distribution of transcripts using PaGeFinder (PDF 8 kb)

Additional file 2: **Figure S2.** Melting curves and agarose gel electrophoresis of PCR products of nine candidate genes. (JPEG 1688 kb)

Additional file 3: **Figure S3.** Cycle threshold (Ct) values of nine candidate reference genes across dehydration samples (A) and temperature samples (B). (PDF 194 kb)

Additional file 4: **Figure S4.** Expression stability values(M) of nine candidate reference genes calculated geNorm under dehydration (A), temperature (B) and all conditions (C) respectively. (PDF 99 kb)

Additional file 5: **Figure S5.** Pairwise variation (V) of 9 candidate reference genes. (PDF 104 kb)

Additional file 6: **Table S1.** Pairwise variation (Vn/n + 1) analysis of 9 candidate. (XLSX 10 kb)

Additional file 7: **Figure S6.** Three boxplot graphs representing the pairwise differences in the gene expression values of 9 candidate reference genes under all conditions (A), dehydration stress (B) and temperature stress (C) respectively. (PDF 368 kb)

## Abbreviations

ACT: Actin
CGS1: Cystathionine gamma-synthase 1
CV: Coefficient of variance
DPE2: Disproportionating Enzyme type 2
DPM: Dispersion measure
EF1-α: Elongation factor 1-α
eIF4A: Eukaryotic translation initiation factor 4A
FHP: Fumarate hydratase precursor
FPKM: Fragments per kilobase of exon model per million mapped reads
GAPDH: Glyceraldehyde-3-phosphate dehydrogenase
MAP: Methionyl aminopeptidase
M-value: Expression stability value
PPK: Polyphosphate kinase
SD: Standard deviation
SV: Stability value
TUA: α-tubulin
UBC: Ubiquitin-conjugating enzyme
