# Supplementary figures and images for: Transcriptome-wide identification of optimal reference genes for expression analysis of Pyropia yezoensis responses to abiotic stress
Source: BMC Genomics. 2018 Apr 13;19:251. doi: 10.1186/s12864-018-4643-8 (PMC5899324; doi:10.1186/s12864-018-4643-8)

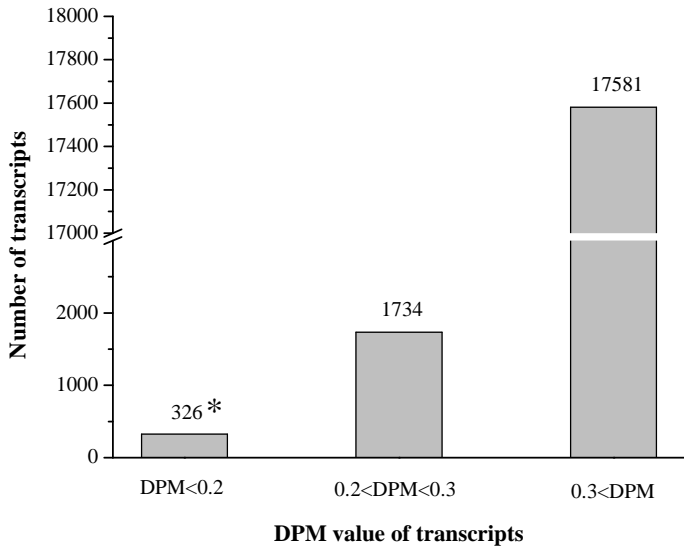

Supplement: Supplementary file 1 — Figure S1. Stability distribution of transcripts using PaGeFinder (PDF 8 kb) [file 12864_2018_4643_MOESM1_ESM.pdf]

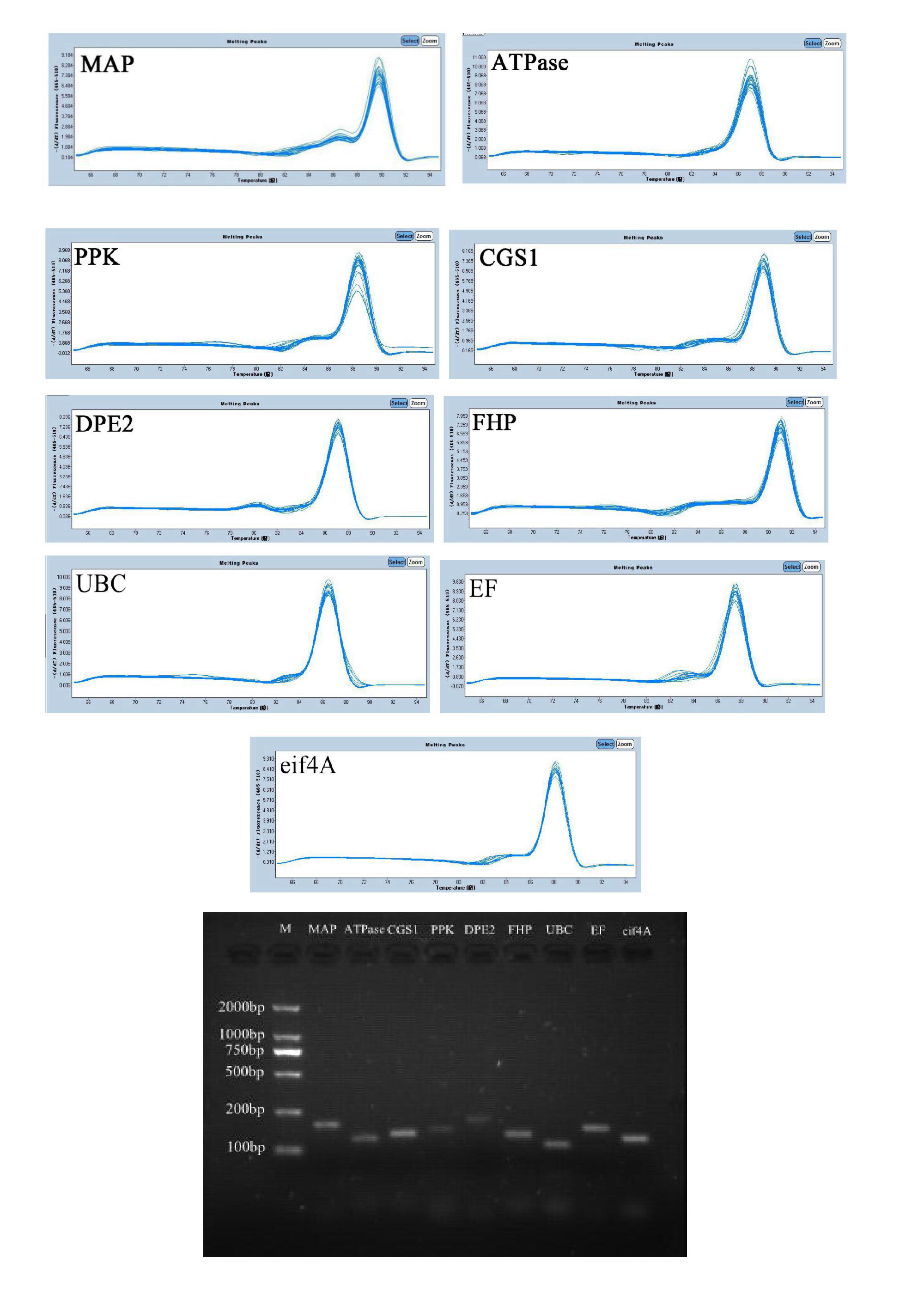

Supplement: Supplementary file 2 — Figure S2. Melting curves and agarose gel electrophoresis of PCR products of nine candidate genes. (JPEG 1688 kb) [file 12864_2018_4643_MOESM2_ESM.jpg]

A

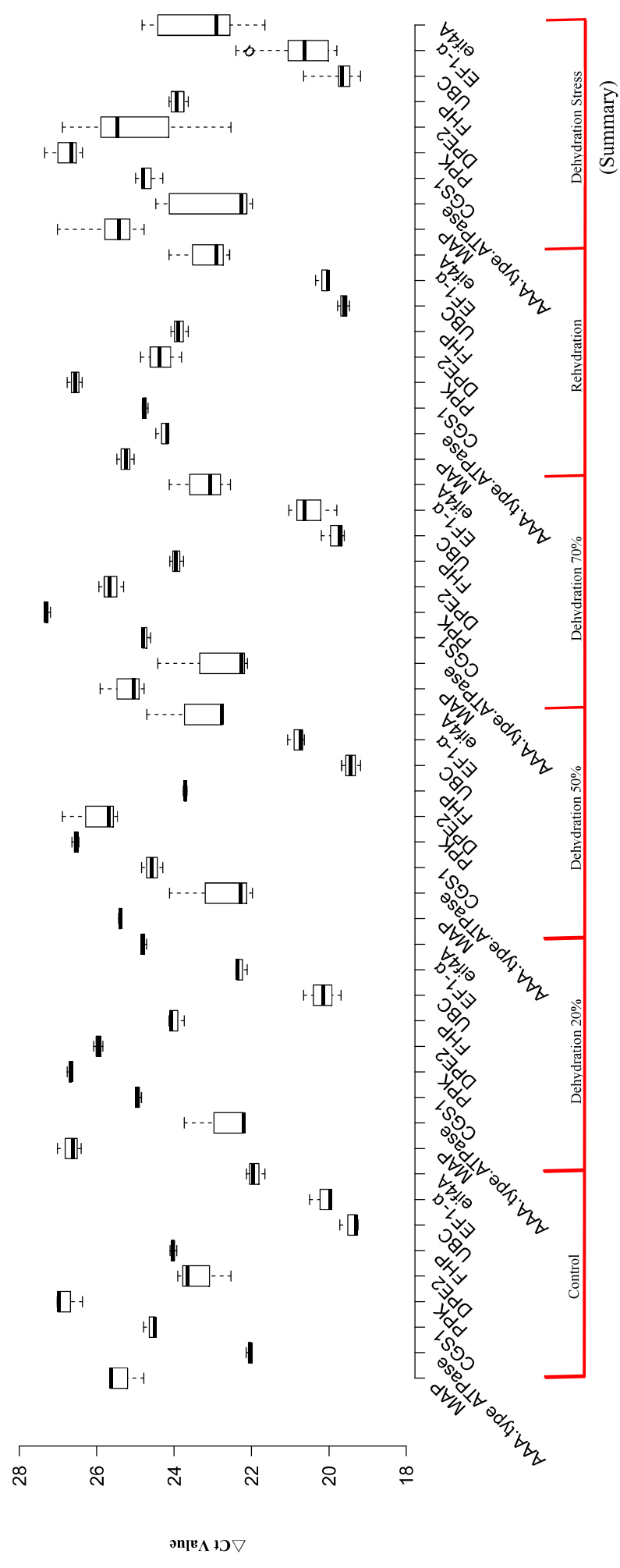

**B**

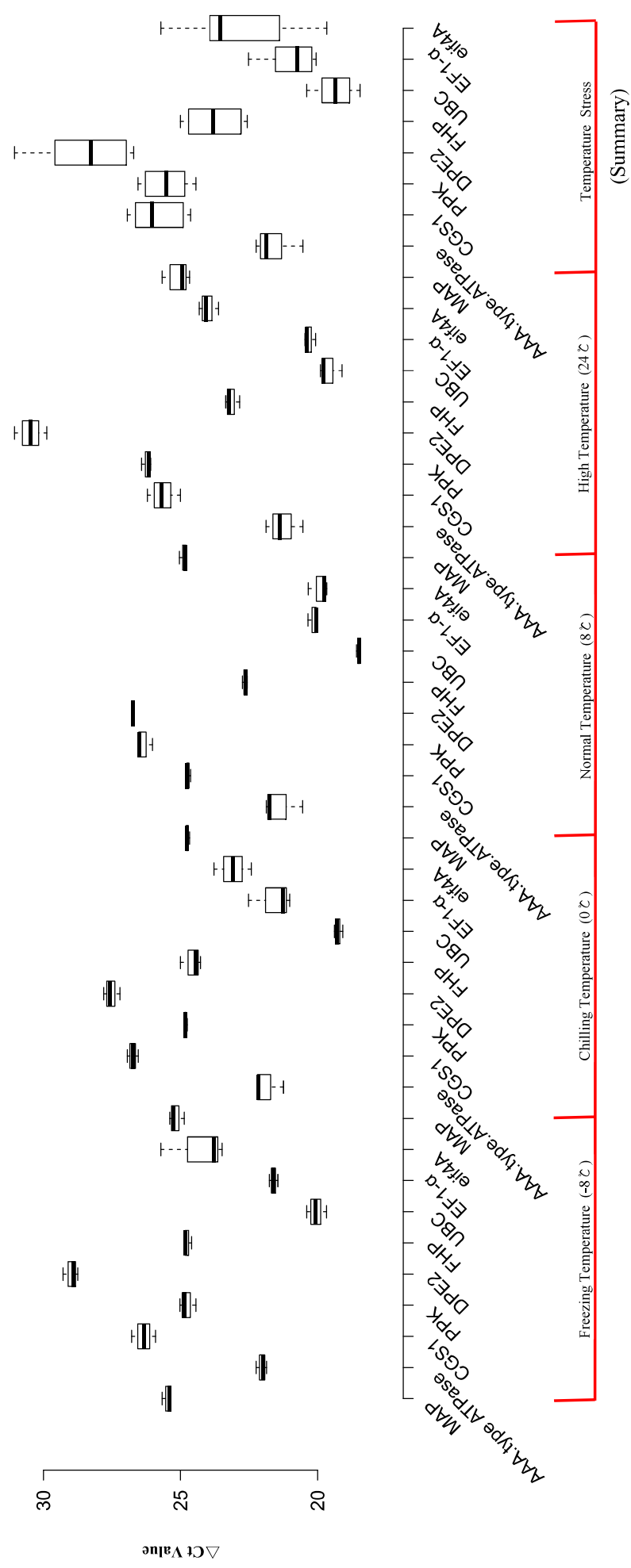

Supplement: Supplementary file 3 — Figure S3. Cycle threshold (Ct) values of nine candidate reference genes across dehydration samples (A) and temperature samples (B). (PDF 194 kb) [file 12864_2018_4643_MOESM3_ESM.pdf]

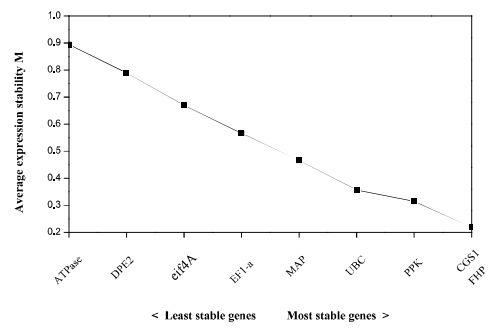

A

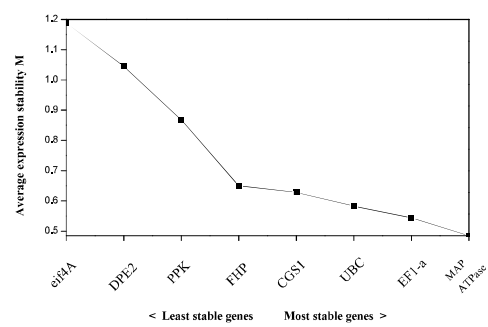

B

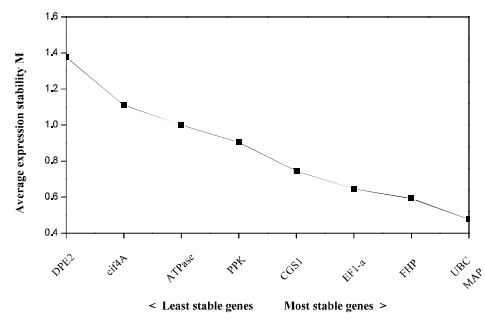

< Least stable genes      Most stable genes >

C

Supplement: Supplementary file 4 — Figure S4. Expression stability values(M) of nine candidate reference genes calculated geNorm under dehydration (A), temperature (B) and all conditions (C) respectively. (PDF 99 kb) [file 12864_2018_4643_MOESM4_ESM.pdf]

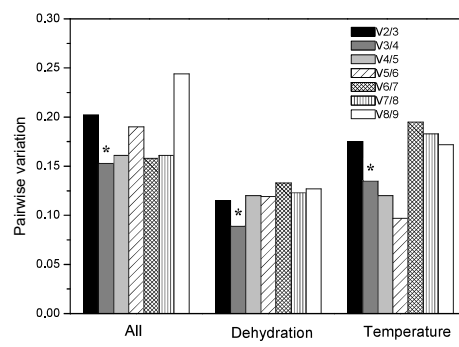

Supplement: Supplementary file 5 — Figure S5. Pairwise variation (V) of 9 candidate reference genes. (PDF 104 kb) [file 12864_2018_4643_MOESM5_ESM.pdf]

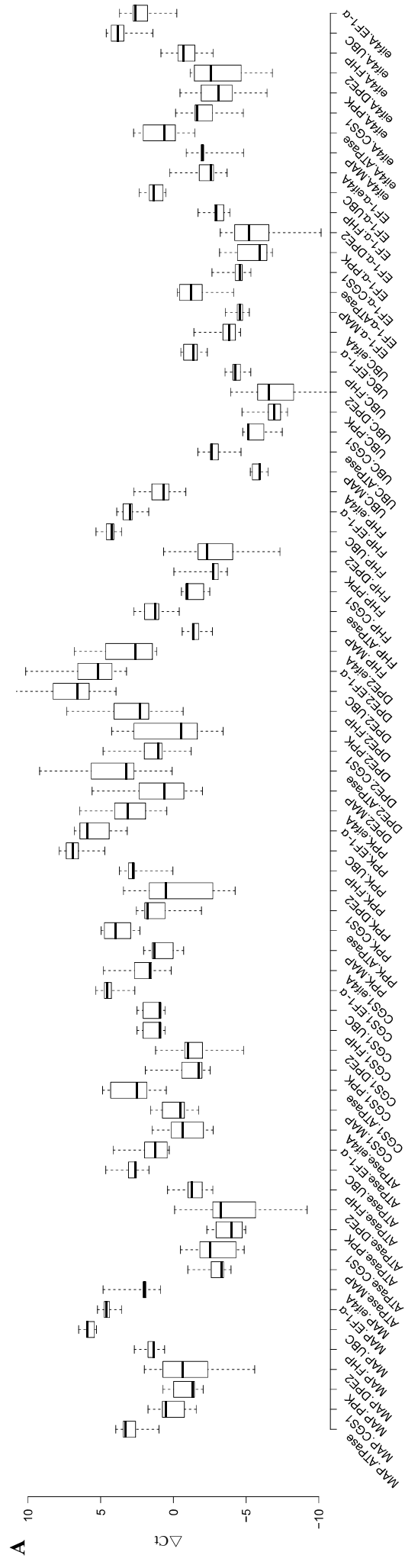

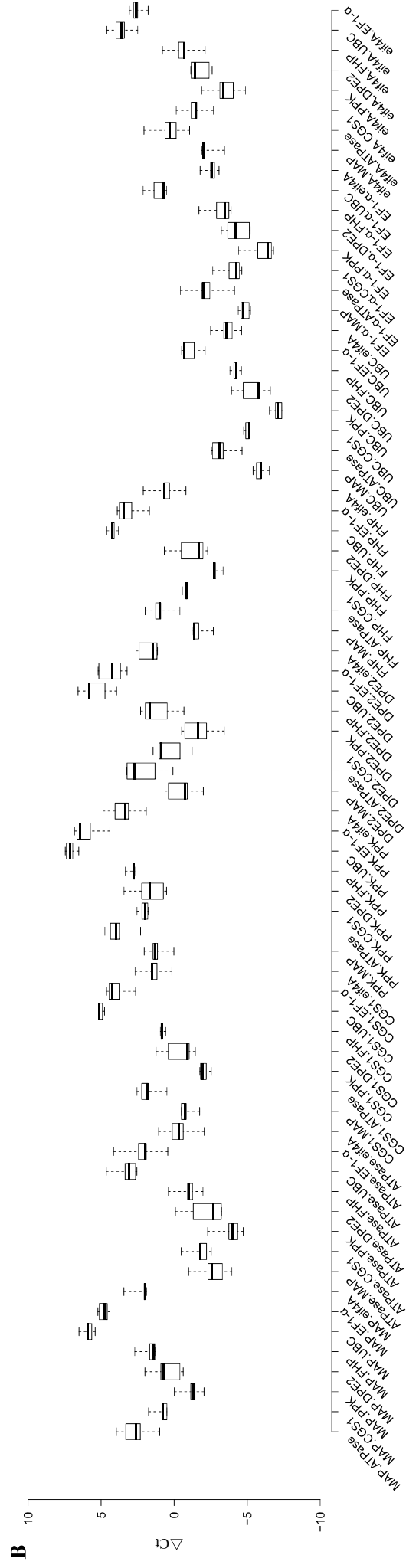

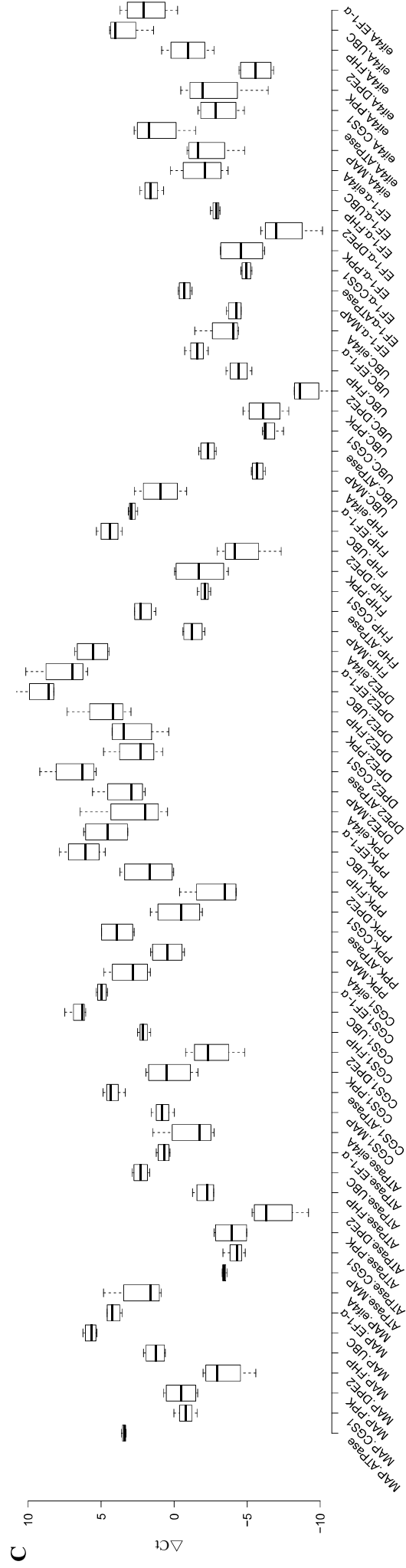

Supplement: Supplementary file 7 — Figure S6. Three boxplot graphs representing the pairwise differences in the gene expression values of 9 candidate reference genes under all conditions (A), dehydration stress (B) and temperature stress (C) respectively. (PDF 368 kb) [file 12864_2018_4643_MOESM7_ESM.pdf]
